# A systematic exclusion induced by institutional ranking in engineering faculty hiring: Introducing a cycle of winners and losers

**DOI:** 10.1371/journal.pone.0275861

**Published:** 2022-12-01

**Authors:** Alireza Ermagun, Jacquelyn Erinne

**Affiliations:** 1 Department of Geography and Geoinformation Science, George Mason University, Fairfax, VA, United States of America; 2 Richard A. Rula School of Civil and Environmental Engineering, Mississippi State University, Mississippi State, MS, United States of America; Beijing University of Technology, CHINA

## Abstract

This study empirically investigates exclusion induced by institutional ranking in engineering faculty hiring and introduces a cycle of winners and losers formed by privileging graduates of high-ranked institutions in the U.S. higher education system. We analyze and visualize academic origin (i.e., institutions faculty graduated from) and destination (i.e., institutions faculty are hired at) of 5,356 tenure-track faculty in four engineering disciplines of Chemical, Civil, Electrical, and Mechanical at the top 20 and bottom 20 of the top 100 engineering institutions according to the 2022 U.S. News & World Report. Our findings indicate that the hiring of engineering faculty in the U.S. higher education system is skewed in favor of graduates from high-ranked institutions, regardless of the discipline. Concerning each engineering discipline, 78% of electrical, 76% of chemical, 71% of mechanical, and 67% of civil engineering faculty of top 20 ranked institutions have academic origins in the top 20 ranked institutions. This hiring practice fosters inequalities by excluding qualified candidates and cementing the ranking system as the sole factor of academic quality. We bring attention to the pitfalls stemming from the exclusion in the U.S. higher education system, including (1) financial resources, (2) faculty and student resources, (3) selectivity and self-selection, and (4) geography. The cascading effect of the ranking practice is the unintended consequence of inaugurating a virtuous and vicious cycle, which creates a cycle of winners and losers that is difficult to break. High-ranked institutions easily dominate and maintain their ascendancy status in the ranking system as benefactors of the virtuous cycle. Low-ranked institutions are entrapped in the vicious cycle that makes it nearly impossible to (1) attract and retain both students and faculty, (2) secure external funding, (3) obtain resources for new programs, and (4) advance engineering research. Unless the U.S. higher education system is intent on squandering talent, confirming the belief that diversity is symbolic, and cementing the ranking system as the sole factor of academic quality, we recommend faculty hiring beyond the standard sociodemographic indicators and academic origins in hiring decisions. A proactive, open-minded, and neutral approach to the faculty selection process void of decision-making based on affinity should be the central tenet of the selection committee.

## Introduction

The U.S. higher education system is no stranger to efforts surrounding Diversity, Equity, and Inclusion (DEI) [1, 2]. From the Civil Rights Act of 1964 [3] and the Executive Order 11246 signed by President Lyndon B. Johnson in 1965 to the enactment of the Title IX Education Amendment Act of 1972 [4], America’s higher education system has grappled with race, gender, age, religious, and national origin discrimination. Despite social advances, movements, and awareness, the problem persists and is exacerbated by creating a more exclusive atmosphere and favoring privileged graduates. High-ranked institutions are inducing a daunting challenge: privileging graduates of high-ranked institutions in the hiring process. The element of “inclusion” has indeed been affected by institutional rankings through a high selectivity of candidates who graduated from high-ranked institutions [5]. Upstream of the faculty hiring process, equal opportunity is dominant in employment advertisements and the hiring pool of higher education institutions. However, downstream, the faculty hiring process is riddled with selection biases of known and unknown criteria and hegemony that further skew the resulting outcome in favor of a candidate from a high-ranked institution.

Selection biases, appearing in the form of institutional, explicit, and implicit, are unfair prejudice in favor of or against a candidate or group held by an individual, committee, or institution in faculty recruitment [6]. They may be expressed when advertising the position announcement or evaluating candidates by incorporating attitudes, beliefs, and stereotypes consciously or unconsciously. Much of the previous research discusses how institutional, explicit, and implicit biases immediately lead to exclusionary or marginalizing practices [7–10] and disproportionately affect minorities [11–14]. By the same token, candidates who graduated from low-ranked institutions are discriminatorily treated in the hiring process of high-ranked institutions. The selection biases are exacerbated by hegemony, where becoming national and international emulation models and state political institutions are coveted by high-ranked institutions [15]. Whether it is the product of hegemony, affinity bias, similarity bias, confirmation bias, halo bias, or perception bias [6], privileging graduates of high-ranked institutions in the hiring process exists and persists [5]. This, to a certain extent, is due to the higher education institution’s rankings with arguable merits [16, 17].

Rankings shape early perceptions of institutions, and homogeneity of thought is achieved to normalize the competition’s discourse, leading to high-ranked institutions [18, 19]. U.S. News & World Report (USNWR), a recognized leader in ranking America’s best colleges, published its first two editions solely on a reputation survey indicator, a measure of how administrators perceive an institution at peer institutions on a peer assessment survey [17]. Although indicators have evolved and involve “quality measures,” the reputation survey indicator and its legacy have lasted long. The 2022 U.S. News & World Report Best Colleges rankings use seventeen subfactors of quality, including graduation and retention rates, social mobility, graduation rate performance, faculty resources, student selectivity, financial resources per student, average alumni giving rate, and graduate indebtedness. Yet, the academic reputation factor has the highest weight [20].

With rankings established as the single norm of excellence, hierarchical systems were established that led higher education institutions to capitalize on reputation, prestige, brand recognition, legitimacy, and networking to achieve upward mobility [21–24]. “The best hiring from the best” is then perceived as an avid quest by higher education institutions to attain the apex of the reputation and prestige pyramid [25]. It gratifies the insatiable appetite of high-ranked institutions for securing external funding, strengthening the network of high-ranked institutions, increasing retention rates, and raising productivity, indicators contributing to calculating rankings and maintaining hegemony. While hiring graduates of high-ranked institutions is deemed to increase research productivity, studies have yet to prove that attendance at high-ranked institutions is associated with performance [26, 27]. Instead, researchers have found that institutions’ attendance correlates with prestigious jobs, high incomes, and upward mobility [28–30].

Faculty hiring from one high-ranked institution into another remains prevalent and raises the question of how well DEI commitments translate to practice. Clauset et al. [31] analyzed the influence of prestige and reputation in the academic system by analyzing 19,000 tenure-track faculty in the computer science, business, and history disciplines in 461 North American departments and school-level academic units. They found that faculty hiring follows a hierarchical structure reflecting profound social inequality, and prestigious institutions hire candidates who completed their doctorate programs from prestigious institutions. In a more recent effort, Kawa et al. [32] conducted a network analysis of 1,918 tenure-track anthropology faculty. They investigated the relationship between faculty placement and resource availability, prestige, and productivity measures. Their outcome showed that a small cluster of institutions was responsible for producing most of the tenure-track faculty, the practice of exclusion through the selection of faculty from a tight-knit peer network of universities, and the importance of citations, awards, and publications for placement and productivity.

The unchartered territory is the hiring practices of tenure-track faculty in high-ranked engineering programs, a critical element of inclusion. Little is known about the faculty hiring network (i.e., who hires whom) in engineering institutions and how it might impact the U.S. higher education system. This study aims to signal the presence of exclusion induced by institutional ranking in engineering faculty hiring and evaluate its adverse consequences on advancing the U.S. higher education system. We investigate the outcome of high-ranked institutions’ hiring practices via an evidence-based approach and present the ramifications of educational advancement in the U.S. higher education system. Therefore, our contribution to the current literature on exclusion in faculty hiring is twofold. First, we examine academic origins (i.e., institutions faculty graduated from) and destinations (i.e., institutions faculty are hired at) of 5,356 tenure-track faculty in the top 20 and bottom 20 of the top 100 engineering programs in the United States across four disciplines of chemical, civil, electrical, and mechanical. We visualize the faculty migration in each discipline separately. The analysis is practical in understanding the faculty migration patterns of the academic origins and destinations, revealing the highest and lowest faculty migration of each top 20 ranked institution. Second, we introduce the virtuous cycle of high-ranked and the vicious cycle of low-ranked institutions to show the complexities and repercussions of rankings on advancing education in engineering institutions. The nuances of the rankings reveal that wealth, socioeconomic status, and financial resources enable high-ranked institutions to maintain their social hierarchy. In contrast, low-ranked institutions cannot secure funding to attract and retain faculty and students, as well as research and develop engineering solutions to problems. In particular, this study examines: (1) whether high-ranked engineering institutions are oversaturated with faculty who graduated from high-ranked engineering institutions in each engineering discipline and (2) the inequality of economic, social, and educational opportunities induced by privileging graduates of high-ranked engineering institutions in the hiring process. This is achieved by a rigorous analysis of comprehensive data extracted from U.S News & World Report (USNWR), institution websites, LinkedIn, National Science Foundation (NSF), U.S Department of Education, and Data USA between October and November 2021.

The remainder of this study is organized as follows. First, we discuss the materials and methods. Second, we present how faculty from high-ranked institutions are hired from high-ranked institutions. Third, we provide an in-depth discussion on the impact of faculty migration patterns dominated in the U.S. higher education system on long-term educational outcomes. Fourth, we conclude the discussion, ponder on potential solutions, report our potential biases, and present potential future research directions.

## Materials and methods

The data collected for our analysis is from six sources: (1) USNWR, (2) institution websites, (3) LinkedIn, Academia.edu, and the dissertation databases, (4) National Science Foundation (NSF), (5) U.S Department of Education, and (6) Data USA. Table 1 summarizes the information of data sources and the data collection methodology. The data collection was performed in two steps. First, we extracted the list of the top and bottom 20 of the top 100 ranked engineering institutions that award doctoral degrees from U.S News & World Report website. Second, we visited each institution’s website and retrieved individual faculty profiles. In the event of missing faculty information on the institution’s websites, we sought other sources, including LinkedIn, Academia.edu, and dissertation databases. Specific to the electrical engineering discipline, the data collection process accounted for the following: (1) for institutions with combined electrical and computer engineering programs and no demarcation in specialty, data includes all faculty in the combined electrical and computer engineering program, (2) for institutions with combined electrical and computer engineering programs and demarcation in specialty, data includes all faculty associated with electrical engineering only, and (3) for institutions with only electrical engineering programs, data includes all faculty in the electrical engineering program. For each tenure-track faculty member in each university, we collected the following information: (1) academic origin (i.e., institutions faculty graduated from), (2) rank of the academic origin, (3) academic destination (i.e., institutions faculty are hired at), (4) rank of the academic destination, and (5) academic ranking (i.e., assistant professor, associate professor, professor). This resulted in 5,356 tenure track engineering faculty from the top 20 and bottom 20 ranked Chemical, Civil, Electrical, and Mechanical Engineering institutions.

**Table 1:** Summary of data sources used for the analysis.

Data Source	Description
2022 Ranking of Best Engineering Schools	Top 20 and Bottom 20 graduate school rankings of the Top 100 engineering institutions were retrieved from U.S. News and World Report (USNWR) [33]. This data set is used in supporting discussion on financial resources (NSF Research funding to institutions and endowments), acceptance rate, retention rate, graduation rates, and student loan default rate.
	Data retrieval: October 3, 2021.
Ranking of Engineering Specific Graduate Programs	“Best Chemical Engineering Programs [34],” “Best Civil Engineering Programs [35],” “Best Electrical Engineering Programs [36],” and “Best Mechanical Engineering Programs [37]” were retrieved from USNWR [33]. The Top 20 and Bottom 20 ranking of the Top 100 engineering institutions of each of the four engineering disciplines are used to determine the faculty’s academic origins and migration patterns.
	Data retrieval: October 3 to October 8, 2021.
Faculty Profile	Information on faculty consisting of the academic origins, the rank of academic origin, academic destination, the rank of academic destination, and academic ranking was directly obtained from the departmental websites where the faculty currently works. Information not directly available on the websites was retrieved from LinkedIn, Academia.edu, and the dissertation databases.
	Data retrieval: October 3 to October 8, 2021.
Research Funding	Data used in assessing the research funding allocated by the National Science Foundation (NSF) was retrieved from the Higher Education Research and Development (HERD) [38] rankings for the fiscal year 2019.
	Data retrieval: November 7, 2021.
Endowment	Data were retrieved from the National Center for Education Statistics [39], under the auspice of the United States Department of Education. This dataset is limited to the top 120 institutions with the largest endowments. We sought information on other institutions from the 2019 annual reports specific to the institutions in question and Data USA [40].
	Data retrieval: November 1 to November 7, 2021.
Institutional Profiles	Information on cost of education, acceptance rates, retention rates, graduation rates, and student loan default rates were directly obtained from Data USA [40] for institutions pertinent to our study.
	Data retrieval: November 1, 2021.

The distribution of 5,356 tenure track faculty over engineering disciplines in the top 20 and bottom 20 of the top 100 engineering institutions is depicted in Table 2. As shown, total faculty hiring in the top 20 ranked institutions is almost twice that of the bottom 20 of the top 100 ranked institutions. Electrical Engineering has the highest number of tenure-track faculty, and Chemical Engineering has the lowest number of tenure-track faculty.

**Table 2:** Distribution of tenure track faculty of top 20 and bottom 20 institutions throughout engineering discipline.

Discipline	Rankings		
	Top 20	Bottom 20	Total
Chemical Engineering	524	299	823
Civil Engineering	755	301	1056
Electrical Engineering	1409	499	1908
Mechanical Engineering	1030	539	1569
**Total**	3718	1638	5356

For each of the top 20 and bottom 20 of the top 100 engineering institutions, we count the total number of faculty and the number of faculty hired by the top 20 ranked institutions. This shows the distribution of faculty hired from the top 20 ranked institutions for all four engineering disciplines. We then pair the origin (i.e., institutions faculty graduated from) and destination (i.e., institutions faculty are hired at) of each faculty member to generate the origin-destination (O-D) migration network. Microsoft Excel, R statistical software, and the Everviz data visualization tool were used to analyze the data and develop the diagrams. We select the bottom 20 of the top 100 engineering institutions as a comparison baseline to examine the difference in the distribution of faculty hired from the top 20 ranked institutions in two not faraway ranking clusters. This selection is neither too far to be concerned about the effects of faculty and financial resources nor too close not to detect any difference in the distribution of faculty hired from the top 20 ranked institutions. We speculate that the disparity, if any, widens with an increase in the rank of institutions.

Before diving into the findings, we reveal our positionality and lens on the data collection and analysis. At the beginning of the study, the first author was a tenure-track faculty of Richard A. Rula School of Civil and Environmental Engineering at Mississippi State University, ranked 121 in the 2022 U.S News Best Colleges Rankings. He received his Ph.D. in Transportation Systems Engineering from the University of Minnesota, ranked 17 in the 2022 U.S News Best Colleges Rankings. He has only pursued academic positions and trained in three national and international programs, including Mississippi State University, Northwestern University, the University of Minnesota, and the University of New South Wales. The themes of justice and equity are threads woven into his research. He has explored historical causes of inequity and exclusion in his discipline and considers diverse points of view, intersectionality analysis, inclusionary themes, and pluriverse in research and practice. At the beginning of the study, the second author was a Ph.D. Candidate of Richard A. Rula School of Civil and Environmental Engineering at Mississippi State University. Her passion for Science, Technology, Engineering, and Math (STEM) has her engaged in her community, where she teaches personal and academic enrichment classes, reviews STEM grants, and reviews and judges STEM projects on a local, regional, national, and international level. Both authors dedicated their time and expertise throughout the 2019–2021 academic year without financial support to signal the presence of exclusion induced by institutional ranking in engineering faculty hiring. This was a collaborative effort with hours of discussions guided by our collective cultural knowledge and expertise.

We believe DEI is multiplexed and challenging to navigate, albeit it is a topic that often appears rudimentary. DEI is subjective and dynamic by human nature as it relies upon individuals’ sentiments, experiences, awareness levels, and exposures. The definitions of DEI vary across the strata of individuals, which makes the measures vary across individuals (e.g., inclusion to one individual is an exclusion to another individual). Although the views expressed here are primarily those of the first author, in the pleasure of discovering and finding things out, we end up with increased awareness levels that often lead to resolve.

### Findings

We find that the top 20 ranked institutions hire an average of 74% of their faculty from the top 20 ranked institutions, while the bottom 20 of the top 100 ranked institutions hire an average of 39% from the top 20 ranked institutions. Specific to the engineering disciplines and for the total number of faculty in the top 20 ranked institutions, 76% of chemical, 67% of civil, 78% of electrical, and 71% of mechanical engineering faculty are hired from the top 20 ranked institutions. In contrast, for the bottom 20 of the top 100 ranked institutions, 34% of chemical, 49% of civil, 29% of electrical, and 45% of mechanical engineering faculty are hired from the top 20 ranked institutions.

**Fig 1:**
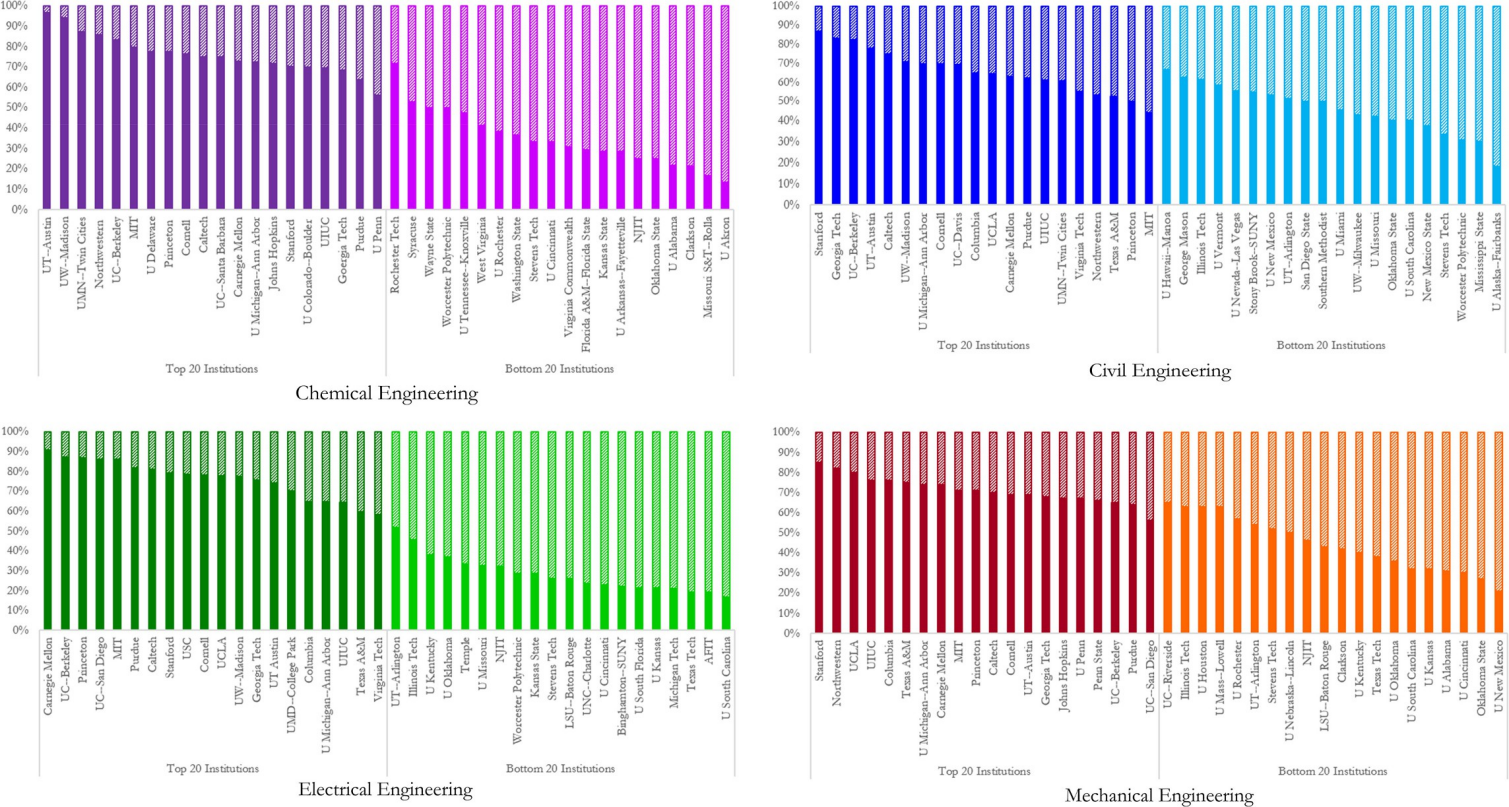
Distribution of faculty hires from top 20 ranked institutions over the top and bottom 20 ranked institutions. (A) Chemical Engineering, (B) Civil Engineering, (C) Electrical Engineering, and (D) Mechanical Engineering. Solid colors represent the share of hires from the top 20 ranked institutions, and the shaded color is representative of the share of hires not from the top 20 ranked institutions.

Fig 1 is a snapshot of the distribution of faculty hired from the top 20 ranked institutions throughout the engineering disciplines for the top 20 and bottom 20 of the top 100 ranked institutions. The reader is referred to S1 Table for the raw data. Three observations are discerned. First, we notice a distortion in the hiring patterns of high-ranked institutions. They hire a larger share of faculty with academic origins associated with similarly ranked networks of high-ranked institutions. This observation is made across the four engineering disciplines. Second, amongst the four disciplines, the chemical and electrical engineering hiring processes depict the most skew in faculty hiring from high-ranked institutions. Faculty in the top 20 ranked chemical engineering programs make up 76% of hires from top 20 ranked institutions. In the electrical engineering discipline, this percentage is 78%. In addition, the lower-ranked institutions in the chemical and electrical engineering program have the least share of faculty hired from high-ranked institutions, with 34% and 29% from the top 20 ranked institutions, respectively. Third, civil and mechanical engineering disciplines have a larger faculty share in the lower-ranked institutions with academic origins from the top 20 ranked institutions.

Specific to the institutions in each engineering discipline, we observe that in the chemical engineering discipline, the University of Texas—Austin has the largest share of faculty (96%), and the University of Akron hires the least share (13%) of faculty from the top 20 ranked institutions. Rochester Institute of Technology is in the bottom 20 of the top 100 ranked institutions attracting 71% faculty share from the top 20 ranked institutions. This share of faculty hires is higher than six of the institutions ranked in the top 20. Concerning the civil engineering discipline, Stanford hires the largest share (87%) of its faculty, and the University of Alaska—Fairbanks employs the smallest share of faculty from the top 20 ranked institutions. We also observe that approximately 50% of the bottom 20 of the top 100 ranked institutions have a similar share of faculty hiring from the top 20 ranked institutions compared to 50% of the top 20 ranked institutions. In the electrical engineering discipline, Carnegie Mellon University has the largest share (91%). The University of South Carolina has the least faculty share with academic origins in the top 20 ranked institutions. In association with the mechanical engineering discipline, Stanford University employs 85% of its faculty from the top 20 ranked institutions. The University of New Mexico hires the least faculty from the top 20 ranked institutions.

**Fig 2:**
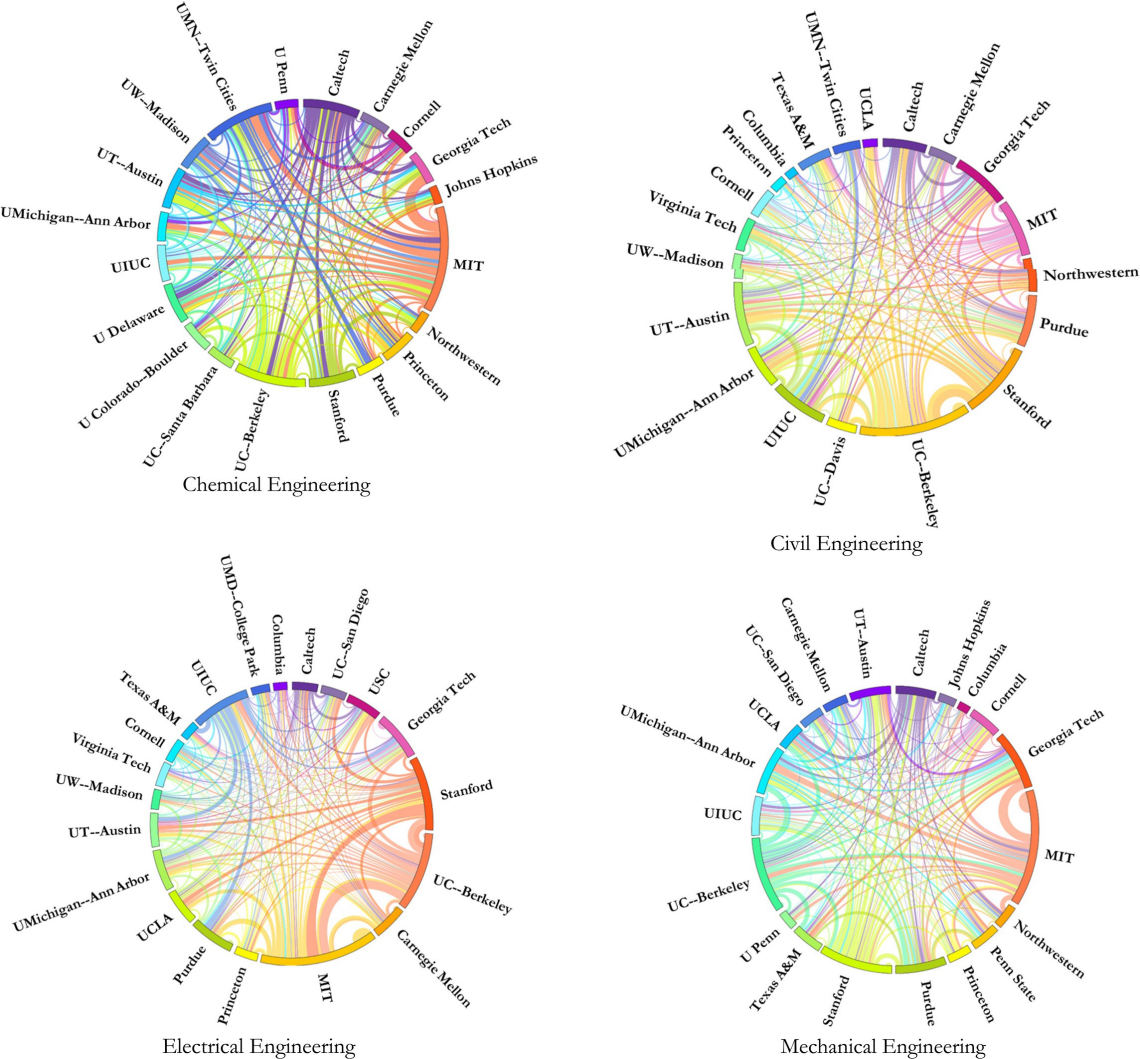
Migration diagram of top 20 ranked institutions. (A) Chemical Engineering, (B) Civil Engineering, (C) Electrical Engineering, and (D) Mechanical Engineering.

Understanding that the top 20 ranked institutions hire the most faculty from the top 20 institutions, we reveal in Fig 2 the highest and lowest faculty migration in each engineering discipline. We generated an O-D pair for only the faculty members who graduated from top 20 ranked institutions and are currently holding tenure-track positions in top 20 ranked institutions and weight the O-D pair that shows the highest and lowest faculty migration in each engineering discipline. Massachusetts Institute of Technology produces and attracts the largest faculty share from the top 20 ranked chemical, electrical, and mechanical engineering institutions. The University of California—Berkeley produces and attracts the most faculty from the top 20 ranked institutions for the civil engineering program. Johns Hopkins University produces and draws the least share of faculty from the top 20 ranked institutions in the chemical engineering discipline. Columbia University is associated with the smallest production and attraction of faculty from the top 20 ranked institutions for the civil, electrical, and mechanical engineering disciplines.

**Fig 3:**
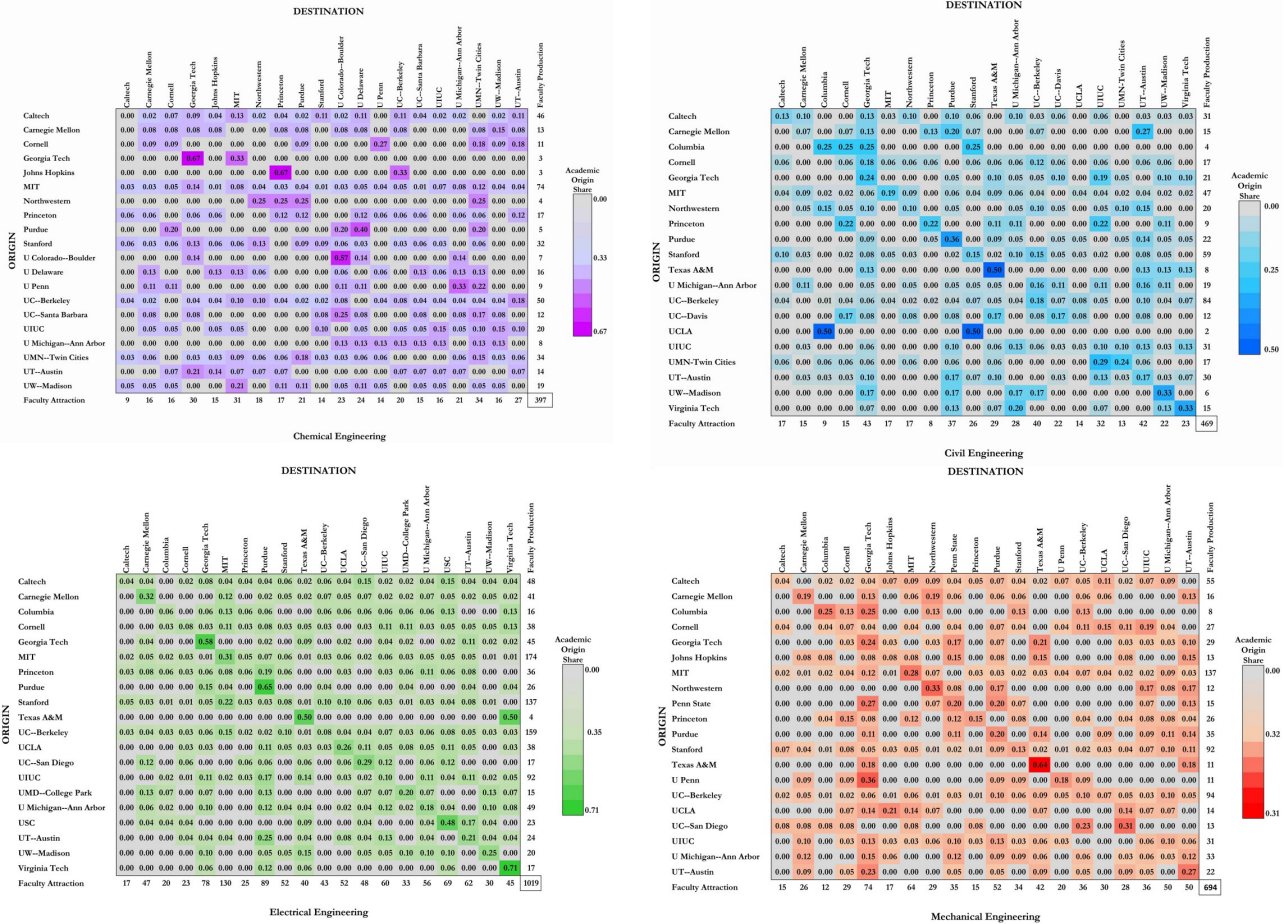
Origin-destination matrix of faculty graduated from top 20 and hired by top 20 ranked institutions. (A) Chemical Engineering, (B) Civil Engineering, (C) Electrical Engineering, and (D) Mechanical Engineering.

To better comprehend more of the migrations of faculty from one top 20 ranked institution to another top 20 ranked institution, we present the origin-destination matrix in Fig 3. To create the heatmaps, we weight the O-D pair of faculty members with academic origins and currently working in the top 20 ranked institutions, sum the weights, and divide each O-D pair weight by the sum of the weight to get the share of faculty produced and faculty attracted. Depicted are the top 20 ranked institutions that produce the fewest graduates and the top 20 that attract the most graduates from the top 20 ranked institutions for each of the four engineering disciplines. Faculty whose information about academic origin was not readily available in any search sources defined in the data collection section were omitted from the assessment. This includes 1 Chemical Engineering faculty, 6 Civil Engineering faculty, 5 Electrical Engineering faculty, and 46 Mechanical Engineering faculty. The origin represents where faculty completed their doctorate program, known as “faculty production.” The destination represents the institution where the faculty is currently employed, known as “faculty attraction.” Massachusetts Institute of Technology produces the most faculty in the chemical, electrical, and mechanical engineering programs. The University of California—Berkeley creates the most faculty in civil engineering programs in the top 20 ranked institutions. It can be deduced that institutions that produce the most faculty are also the most ranked, and those that produce the fewest faculty are ranked lower in the top 20 rankings. Students who graduated from the top 20 ranked engineering programs are mainly attracted by the University of Minnesota—Twin Cities in chemical engineering, Georgia Tech in civil and mechanical engineering, and Massachusetts Institute of Technology in electrical engineering disciplines. As an extension of the O-D matrix, we graphically show the migration patterns of faculty academic origins from the top 20 ranked institutions to the destination where the various engineering programs hire them.

## Discussion

**Fig 4:**
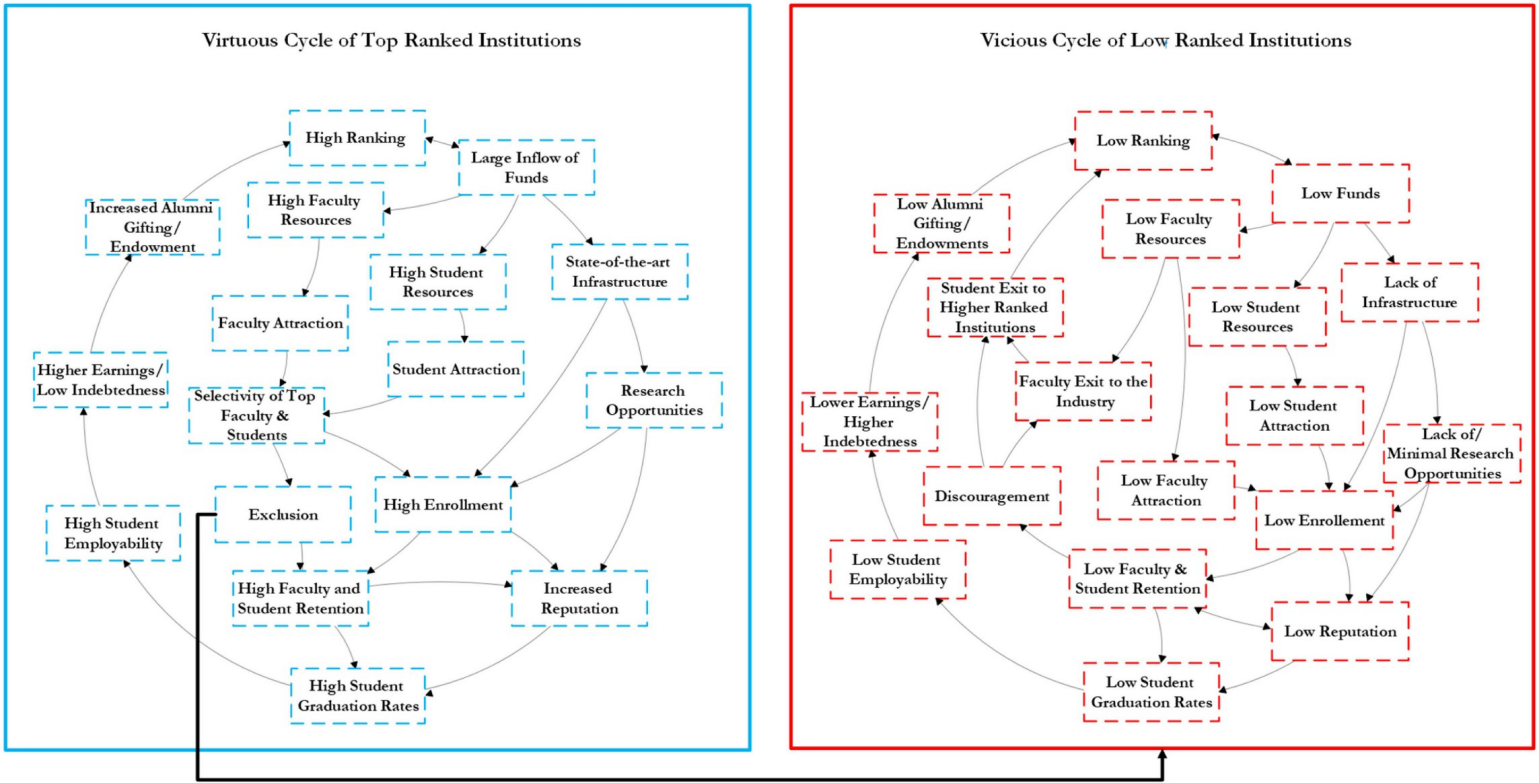
The virtuous cycle of high-ranked institutions and the vicious cycle of low-ranked institutions.

Our analysis revealed that engineering schools in high-ranked institutions are oversaturated by tenure-track faculty hired from high-ranked institutions. This heavy concentration adversely affects advancing education across the United States. The quest for domination has shown that, in the words of Gioia and Corley [41], “all things wrong with the rankings matter considerably less than the plain fact that the rankings matter.” The outcomes of hiring faculty by high-ranked institutions from predominantly high-ranked institutions create a “virtuous cycle” of favorable results for the institution, faculty, and students. This virtuous cycle is further strengthened by the forces of financial resources, wealth, socioeconomic status, affinity, social hierarchy, and hegemony, which tethers together the convoluted sequences of events. Low-ranked institutions are ensnared in a series of detrimental events that keep them in a “vicious circle” with little to no intervention or support, which is paramount in advancing future generations of engineers. Another way of describing the virtuous cycle of high-ranked institutions and the vicious cycle of low-ranked institutions is the concept of the “Matthew Effect” whereby the elite receive disproportionate credit and resources [42]. Fig 4 depicts the virtuous and vicious cycles of high- and low-ranked institutions.

Graduates of low-ranked institutions barely stand a chance to be hired by high-ranked institutions, even in situations where they are qualified and capable of delivering on teaching, research, and service. The hiring process presents the notion that hiring is based on equal opportunity and compliance with federal law, where any candidate can apply for any faculty position. Beyond the receipt of applications from a vast pool, however, no law governs how a candidate is selected. In the absence of a defined metric, the selection process is subjective in nature and biased. One can only begin to imagine the selection process as a “black box.” [43] Wright and Vanderford [44] drew attention to the need for transparency and highlighted university ranking as a powerful predictor in faculty search, further maintaining the robust social rank system. Our study is not associated with the intricacies of the selection process. Instead, we showed a solid and dominant social hierarchy faculty hiring system in the U.S. higher education system. We now parse primary concerns induced by privileging high-ranked graduates in institutions’ hiring process, which introduces a cycle of winners and losers in academia.

### Financial resources

The inflow of financial resources is essential to the operations of any institution. Sources of funds into institutions include, but are not limited to, the federal government, state government, local government, institution funds, businesses, nonprofit organizations, and endowments. The funds support building infrastructure, innovation through research, faculty resources for teaching and research, and student resources critical for learning. The reader is referred to S2 Table for information on the funding sources.

America’s research structure changed post World War II [45], moving from individual and institutionalized research to a government-university partnership that sought to engage universities in advancing the goals of federal agencies and national interests not limited to national security, economic development, and health. Through this partnership, America’s congress allocates government resources to institutions through various governmental agencies. The National Science Foundation (NSF) [46] is the primary government agency responsible for promoting science and engineering education in educational settings. NSF is the funding source for a quarter of the total government budget for basic research and education in all scientific and engineering disciplines. By the competitiveness associated with research grants to support students, faculty, tools, and infrastructure, high-ranked institutions are favored to secure a larger share of funding. This leaves low-ranked institutions in no position to thrive.

One main observation is the inequitable resource allocation to high-ranked and low-ranked institutions. Out of $654.3 billion issued in total research and development (R&D) expenditures in the U.S. higher education institutions between 2008 and 2017 [47], 24% was collected by the top 20 ranked institutions, while only 6% was obtained by the bottom 20 of the top 100 ranked institutions. More tragic is collecting 8% of the funds by eight Ivy League institutions and a mere 2% of the fund allocation to the lowest 20 institutions in the entire engineering programs designated by USNWR. According to the most recent published data [38], the top 20 ranked institutions of pertinence to our study received six times the funds allocated to the bottom 20 of the top 100 ranked institutions. Specific to the individual institutions, John Hopkins University (top 20) received 243 times more R&D funds than Embry-Riddle Aeronautical University (bottom 20) as the lowest recipient. This disparity is abysmal, considering the United States has 394 public and non-profit 4-year institutions with research classifications [48] ranging from “not high level” to “very high level of research” activities. Funding of such a minuscule amount to lower-ranked institutions makes it impossible for students to be competitive and innovate due to a lack of access to tools, physical or operational infrastructure, and faculty to conduct research. On the basis that the funding sources of R&D expenditures are primarily from taxpayers, this allocation is inequitable, excluding lower-ranked university students and faculty from contributing to discoveries paramount to furthering the U.S. national goals in health, economic development, and security.

Endowments from donors, another well-known means of financial inflow into institutions, support student financial aid, innovative academic programs and fields, technological improvements, faculty positions, research and development, and infrastructure. These endowments are also mediums to serve future generations, invest in the institutions’ future, and maintain the long-term operations of the school. The top 20 ranked institutions command endowments of $144.5 billion, and the bottom 20 of the top 100 ranked institutions received $9.9 billion [39, 40]. In general, the top 20 ranked institutions received fifteen times more funds in the form of endowments than the bottom 20 of the top 100 ranked institutions. The financial strength between the largest recipient (Stanford University) in the top 20 and the least recipient (Florida A&M) in the bottom 20 rank is 315 times, giving Stanford University the advantage of investing more in their students’ future and ultimately reducing the cost of students’ education. Stanford University’s investment per student through endowments is $1.7 million based on undergraduate enrollment data compared to a scanty $10,000 investment per student at Florida A&M.

### Faculty and student resources

Faculty and student resources are made available through various funding resources. We found that the top 20 ranked institutions can hire approximately two and a half times more faculty than the bottom 20 of the top 100 ranked institutions. The electrical and mechanical engineering programs have sufficient funds to hire three times more faculty in the top 20 than the bottom 20 of the top 100 ranked institutions. This faculty hiring is two times more in the chemical and mechanical engineering disciplines. Student enrollment exhibits a similar pattern as the top 20 ranked institutions attract fourteen times more students than the lower 20 ranked institutions. Lower enrollments result in less financial resources in lower-ranked institutions. The vast faculty and student resources lead to favorable conditions for the top 20 ranked institutions—higher student retention rates, higher graduation rates, and lower student loan defaults than the bottom 20 of the top 100 ranked institutions. On average, the retention, graduation, and student loan default rates are 96%, 90%, and 2%, respectively, for the top 20 ranked institutions. There is a stark difference compared to the bottom 20 of the top 100 ranked institutions, where the average retention, graduation, and student loan default rates are 85%, 66%, and 30%, respectively. Previous studies support that graduates of high-ranked institutions benefit from better access to the workforce, top jobs, top incomes, and upward mobility [28–30]. The legacy of rankings supports this stemming from resource allocation.

### Selectivity, self-selection, and exclusion

If not all, most high-ranked institutions attest to taking on sensitive and complex topics on diversity, equity, and inclusion and stressing the need for DEI as part of their diversity recruitment plans. If DEI is premised on accommodating differences, ensuring impartial and fair processes from beginning to end, and then privileging graduates of elite institutions in the hiring process cements the logic to exclusion [43]. In this case, the logic of exclusion alludes to intentionally excluding the candidate pool in the initial screening process that does not fall under the auspice of the top 20 ranked, Ivy League, Tier 1, and top globally ranked institutions. Judging applicants by their institutional ranking and affinity is not a strange phenomenon for faculty serving on academic search committees. Through the highly selective process of attracting students and faculty from high-ranked institutions, the purpose of inclusion has been defeated. Graduates of lower-ranked institutions are not afforded the opportunities to become faculty in higher-ranked institutions, even when qualified. We see here that the high selectivity of top students and faculty from similarly ranked institutions leads to exclusion.

Similarly, the process of self-selecting faculty who graduated from the same top-ranked institution excludes other schools of thought in the academic sphere, which gives rise to inbreeding. The faculty production and attraction matrix in Fig 3 confirms this axiom. For example, the Massachusetts Institute of Technology produced and retained the largest share of faculty in the electrical engineering discipline, which is indicative that the electrical engineering department maintains the school of thought rooted in their institution. This same sample can be expanded to the top 20 ranked institutions. In the electrical engineering program, 78% of the faculty are produced by the top 20 ranked institutions. For chemical, mechanical, and civil engineering disciplines, faculty hires from the top-ranked institutions are 75%, 71%, and 67%, respectively. The diversity of thought is therefore hampered and excludes other thought processes. However, pursued to strengthen the social network and is deemed to acquire reputation and prestige as social capital [25, 49], this practice might cause a closed system due to the inability to incorporate new and fresh voices and ideas [50]. Isolationism leads to an unhealthy system with unstable and unsustainable characteristics [50]. Advocates who favor hiring their doctorate graduates consider this practice reaping the rewards of their investment in the student through educational funding (e.g., research assistant, teaching assistant, scholarships), research and publications, professional development, and other training. Another reason is the appeal of familiarity of their students as their work is known and they have proven themselves, as inviting “outsider candidates” for a short interview does not offer a complete insight into their teaching, research, and personal characteristics. The issue of self-selection, however, is a bi-directional issue that has institutions wanting graduates from high-ranked institutions and graduates from high-ranked institutions intentionally seeking employment dominantly in high-ranked institutions. With a supply of top 20 ranked graduates and low demand for faculty positions in top 20 institutions, the chance for doctorates from low-ranked institutions to have a voice in high-ranked institutions is even lower.

In the competitive field of faculty applications, academic faculty origin as a selection criterion creates a skewed system that automatically discourages well-qualified candidates from participating in the application process. Knowing the chances of being selected as faculty in a high-ranked institution is extremely low, potential candidates refrain from investing their time and effort in the rigorous application process of prepping cover letters, curriculum vitae, a summary of research, teaching philosophy, and diversity advancement plan. This discouragement leads to seeking alternative employment in lower-ranked institutions where DEI is practical or to pursuing industry openings.

### Geography

The geography of the top 20 ranked engineering institutions is concentrated in 10 out of the 50 states, with 65% of the top 20 ranked institutions in the Northeast and Western regions and 20% in the Midwest. Both the Southeastern and Southwestern regions have the least share. Institutions in the Northeast and Western regions are enormous benefactors of R&D government funding and endowments, attracting and retaining a centralized U.S. workforce to these regions and stagnating engineering development and advancement across other country areas. California alone has five institutions on the top 20 ranked engineering list and is listed as the best state for engineers to live and work [51]. The educational and professional concentration of engineers in these locations degenerates’ the science, technology, engineering, and mathematics (STEM) field that excludes the diversity of thoughts from 40 other states. This is disadvantageous to future graduates and engineers who are unaware of engineering issues, applications, and solutions unique to varying U.S. geographical areas.

## Conclusion

Although not originally intended to be used as a measure of academic quality, traditional methods of ranking institutions have dominated higher institutions in their quest for reputation, prestige, wealth, and social status. The process of rankings has “conferred gate-keeper status on elite educational institutions because they are perceived as having the capability to boost one’s status relative to another [52].” Rankings have further created a “positional arms race” [53], which establishes the position of institutions and success by their ability to attract quality students and spend significant funds per student. The cascading effect of the ranking practice is the unintended consequence of inaugurating a virtuous and vicious cycle, which creates a cycle of winners and losers that is difficult to break. High-ranked institutions easily dominate and maintain their ascendancy status in the ranking system as benefactors of the virtuous cycle. More definitively, the virtuous cycle establishes barriers to entry [35] in the U.S. higher education system that is detrimental to the competitive landscape of advancing education in engineering institutions. Low-ranked institutions are entrapped in the vicious cycle that makes it nearly impossible to (1) attract and retain both students and faculty, (2) secure external funding, (3) obtain resources for new programs, and (4) advance engineering research. This affects the low-ranked institutions’ ability to rise in the ranking system and makes it practically impossible for low-ranked institutions to recover in rank. Faculty and students from low-ranked institutions are then excluded from playing a significant role in advancing education in engineering institutions.

The insatiable appetite of higher education institutions for reputation, prestige, wealth, and social status has begotten an obsession with the academic rankings, which has, at times, led to lying and cheating. A few examples of intentional and unintentional falsification are the University of California—Berkeley [54], the University of Southern California [55], Columbia University [56], and Temple University [57]. The University of California—Berkeley misreported their alumni giving rate, an indicator accounting for 5% of the USNWR ranking calculation, from 2014 to 2019. Although the USNWR moved them to the “Unranked” category in its 2019 edition, there were neither further investigations nor punishment. The University of Southern California cheated by excluding the information on the doctor of education students to achieve a higher ranking from 2013 to 2021. Columbia University landed 2^nd^ in the 2022 USNWR rankings by fabricating faculty, instructional spending, and class size data. Temple University systematically lied to the USNWR to boost its business school rankings. Is the blame placed on the U.S. higher education system, institutions, presidents, provosts, deans, college rankings giant, and hierarchical systems? That is a rhetorical question.

Traditional methods of ranking institutions are also void of creating economic mobility for underserved students. In a most recent report, Itzkowitz [58] posited that college rankings are not reflective of the purpose of our higher education but rather reproduce existing inequalities in higher education that rewards wealthy and selective institutions. He proposed Economic Mobility Index (EMI) as a rating system assessing institutions based on their high acceptance of students from low and moderate backgrounds and providing these students with a strong return on their investment. He concluded that non-traditionally ranked institutions serve the underrepresented students more than top-ranked institutions. In instances where high-ranked institutions score high on the EMI, it is for reasons that the admitted low-income students are high achieving with institutional support from a wide range of institutional resources. Two other powerful examples of a counter-hegemonic ranking system are “Social Mission Score” and the “Estudio Comparativo de Universidades Mexicanas.” [15] Although any ranking system eventually leads to establishing hierarchical systems, it should be noted that its existence is inevitable [17]. With this in mind, universities should consider the power of diversifying whom they employ to continue to compete in the global educational sphere. As education becomes more of a global commodity, by only hiring from a small pool of universities they are cutting themselves off from a literal world of talent who will be hired by other universities, which will bolster their standings [59].

In presenting the virtuous and vicious cycles, we showed how low-ranked institutions do not stand a chance in gaining the wealth and resources to serve the students as the wealth is concentrated in high-ranked institutions. The effect is an expansion of exclusion for which the institutions actively seek to resolve. A starting point to not privileging graduates of high-ranked institutions in the hiring process is to recognize that the ranking system is not the lone factor of academic quality. While this is a well-known private truth within educational settings, higher education institutions are eager to maintain the traditional legacy ranking systems that give them the advantage of acquiring funds through endowments and research. Consequently, only high-ranked institutions are privy to securing wealth and resources that provide an advantage in servicing students, ultimately creating a system of winners and losers. Unless the U.S. higher education system is intent on squandering talent, confirming the belief that diversity is symbolic, and cementing the ranking system as the sole factor of academic quality, we recommend hiring beyond the standard sociodemographic indicators and the academic origins in the faculty hiring decision. However, we neither make the case against the decisions of high-ranked institutions to hire from high-ranked institutions nor the self-selection of high-ranked graduates to migrate to high-ranked institutions.

A proactive, open-minded, and neutral approach to the faculty selection process void of decision-making based on affinity should be the central tenet of the selection committee. We suggest the following actions while acknowledging the exclusion induced by institutional ranking as a wicked problem. Our suggestions are then not right or wrong but are better or worse. First, institutions should come to a shared understanding of diversity and inclusion and how hiring new voices and fresh ideas, regardless of the academic origin, relates to their vision or mission. Second, departments should seek administrative support from the office for equity, diversity, and inclusion to identify and share best practices. If the academic origin of candidates is tracked throughout the faculty hiring process, barriers of exclusion from past experiences can be assessed, and guidelines can be modified to counteract hiring bias. Third, departments also should select search committees and particularly the chair of search committees from members who are known diversity advocates. Search committee members, for example, should include faculty who graduated from low-raked institutions or who chaired searches in which faculty with low-raked institutions’ academic origin were hired to assist committees. Follow-up interviews should then be conducted with candidates who were not contacted or interviewed and were not offered to augment the element of inclusion in the hiring process. Fourth, human resources are encouraged to exclude the academic origin of candidates from application materials forwarded to search committee members. Although it is time-intensive, it helps reduce implicit bias and forces search committee members to evaluate the originality and quality of materials. It levels the playing field as candidates’ alma matter is excluded from the hiring equation. Fifth, human resources are encouraged to ask search committee members to articulate and document their inclusion and exclusion criteria. Codifying selection criteria and establishing rubrics for assessment facilities fair evaluation of candidates if employed through all stages of recruitment and used to guide discussions.

As we pondered on exclusion induced by institutional ranking in engineering faculty hiring, it is proper to self-reflect on our potential biases and partial views. While we selected materials and methods carefully and reported findings from a neutral point of view, bias is naturally woven into our method and assessment. The first potential bias relates to our data. For each institution, we compared the number of faculty hired from top 20 institutions with the number of faculty who are hired from not top 20 institutions. This might overestimate or underestimate the exclusion of candidates who graduated from low-ranked institutions in engineering faculty hiring of high-ranked institutions. Our findings are overestimated and exaggerated if the number of doctorates awarded in top 20 institutions dominates the U.S. faculty job market, as their predominance is also expected in faculty hiring without institutional ranking discrimination. However, as the number of doctorates who graduated from top 20 institutions is less than those who graduated from not top 20 institutions, we underestimated the existing systematic exclusion. This bias is inevitable as the distribution of doctorates awarded over institutions does not represent the distribution of candidates over institutions in the U.S. faculty job market tank. The reason is that nonacademic jobs consume many engineering graduates of high-ranked institutions, and many engineering graduates of low-ranked institutions give up seeking academic jobs due to their low chance of acceptance. This number is unknown. An unbiased solution is to look at the hiring pool of higher education institutions for each advertised position announcement. This necessitates the commitment of higher education institutions to concede inside the “black box” of hiring applications. The second potential bias also relates to our data. We divided higher education institutions into top 20 and not top 20 ranked institutions. This immediately ignores the institutional ranking difference between 21 and 221. An unbiased solution is to represent a cumulative distribution of institutional ranking of academic origin of faculty in each institution while accounting for continuous ranking rather than a dichotomized ranking. This draws a detailed picture of the institutional ranking of the academic origin of faculty hired by high-ranked institutions. It, however, does not change the fact that the U.S. higher education system is skewed in favor of graduates from high-ranked institutions. The third potential bias relates to reporting the virtuous and vicious cycle. This is a simplification of how exclusion induced by institutional ranking in engineering faculty hiring could strengthen the virtuous and vicious cycle. The contributing components of the virtuous and vicious cycle are hardly exhaustive, and the nexus of components are suppositions. This is partially due to the lack of evidence.

Future research could benefit from other aspects not addressed in our study. First, a potential future research avenue is expanding the network analysis of faculty hiring on a broader scale beyond the four engineering disciplines examined in the current research to other science, technology, engineering, and mathematics (STEM) disciplines. Second, our study is limited to the examination of migration patterns of engineering faculty. Further avenues exist to delve into faculty’s individual-level characteristics at varying ranks to understand the disparities. Third, expanding our analysis to encompass non-tenure-track faculty is a potential for gaining insights into the faculty representation in academia compared to faculty with industry experience. This evaluation might also offer revelations that non-tenure-track faculty are not selected using similar criteria as tenure-track faculty. Fourth, data on the number of individuals who applied for the tenure-track faculty position showed a willingness to accept tenure-track faculty positions or declined offers is not publicly available. Such information will offer revelations on the willingness of faculty to work in high-ranked institutions. Overall, these future research aspects warrant further investigation that could advance the dialogue relevant to bridging diversity, equity, and inclusion gaps in the U.S. higher education system.

## Supporting information

S1 Table US news & world report top 100 ranking—Top 20 and bottom 20 ranks and faculty hire from top 20 institutions for four engineering disciplines. (DOCX)

S2 Table All Engineering program rankings and financial profiles. (DOCX)
