# Supplementary material for: A systematic exclusion induced by institutional ranking in engineering faculty hiring: Introducing a cycle of winners and losers
Source: PLoS One. 2022 Dec 1;17(12):e0275861. doi: 10.1371/journal.pone.0275861 (PMC9714811; doi:10.1371/journal.pone.0275861)
Supplement: S1 Table — (DOCX) [file pone.0275861.s001.docx]

**S1 Table. US News & World Report top 100 ranking - Top 20 and bottom 20 ranks and faculty hire from top 20 institutions for four engineering disciplines.**

|  | **Chemical Engineering** | | | |  | **Civil Engineering** | | | |  | **Electrical Engineering** | | | |  | **Mechanical Engineering** | | | |
| --- | --- | --- | --- | --- | --- | --- | --- | --- | --- | --- | --- | --- | --- | --- | --- | --- | --- | --- | --- |
|  | **Rank** | **Colleges** | **Total number of faculty** | **Hired from Top 20 School** |  | **Rank** | **Colleges** | **Total number of faculty** | **Hired from Top 20 School** |  | **Rank** | **Colleges** | **Total number of faculty** | **Hired from Top 20 School** |  | **Rank** | **College** | **Total number of faculty** | **Hired from Top 20 School** |
| **Top 20** | 1 | Massachusetts Institute of Technology | 39 | 31 |  | 1 | University of California--Berkeley | 50 | 41 |  | 1 | Massachusetts Institute of Technology | 157 | 138 |  | 1 | Massachusetts Institute of Technology | 91 | 65 |
|  | 2 | California Institute of Technology | 12 | 9 |  | 2 | Georgia Institute of Technology | 54 | 45 |  | 1 | Stanford University | 63 | 53 |  | 2 | Stanford University | 41 | 34 |
|  | 2 | University of California--Berkeley | 24 | 20 |  | 2 | University of Illinois--Urbana-Champaign | 61 | 38 |  | 1 | University of California--Berkeley | 51 | 45 |  | 3 | California Institute of Technology | 23 | 16 |
|  | 4 | Georgia Institute of Technology | 44 | 30 |  | 4 | Stanford University | 30 | 26 |  | 4 | California Institute of Technology | 21 | 17 |  | 3 | University of California--Berkeley | 52 | 36 |
|  | 4 | Stanford University | 20 | 14 |  | 4 | University of Texas--Austin (Cockrell) | 54 | 44 |  | 5 | Georgia Tech | 106 | 83 |  | 5 | Georgia Institute of Technology | 116 | 78 |
|  | 4 | University of Minnesota--Twin Cities | 39 | 34 |  | 6 | Purdue University--West Lafayette | 61 | 37 |  | 5 | University of Illinois--Urbana-Champaign | 94 | 68 |  | 5 | University of Illinois--Urbana-Champaign | 54 | 41 |
|  | 7 | University of Texas--Austin (Cockrell) | 28 | 27 |  | 7 | Massachusetts Institute of Technology | 40 | 18 |  | 7 | University of Michigan--Ann Arbor | 79 | 58 |  | 5 | University of Michigan--Ann Arbor | 69 | 52 |
|  | 8 | University of California--Santa Barbara | 20 | 15 |  | 7 | University of Michigan--Ann Arbor | 37 | 29 |  | 8 | Carnegie Mellon University | 55 | 50 |  | 8 | Cornell University | 40 | 30 |
|  | 8 | University of Delaware | 31 | 24 |  | 9 | Carnegie Mellon University | 24 | 15 |  | 9 | Cornell University | 36 | 28 |  | 8 | Purdue University--West Lafayette | 90 | 59 |
|  | 10 | Princeton University | 22 | 17 |  | 9 | Virginia Tech | 48 | 26 |  | 9 | Princeton University | 32 | 28 |  | 10 | Carnegie Mellon University | 35 | 27 |
|  | 10 | University of Michigan--Ann Arbor | 29 | 21 |  | 11 | Cornell University | 23 | 16 |  | 11 | Purdue University--West Lafayette | 112 | 93 |  | 10 | Princeton University | 23 | 16 |
|  | 12 | University of Illinois--Urbana-Champaign | 23 | 16 |  | 11 | University of California--Davis | 39 | 27 |  | 11 | University of Texas--Austin (Cockrell) | 90 | 70 |  | 10 | University of Texas--Austin (Cockrell) | 70 | 51 |
|  | 12 | University of Wisconsin--Madison | 17 | 16 |  | 13 | California Institute of Technology | 24 | 18 |  | 13 | University of California--Los Angeles (Samueli) | 70 | 56 |  | 13 | Johns Hopkins University (Whiting) | 26 | 17 |
|  | 14 | Northwestern University (McCormick) | 21 | 18 |  | 14 | Northwestern University (McCormick) | 31 | 17 |  | 14 | Columbia University (Fu Foundation) | 36 | 24 |  | 13 | Northwestern University (McCormick) | 38 | 31 |
|  | 14 | University of Colorado--Boulder | 33 | 23 |  | 14 | Princeton University | 19 | 10 |  | 15 | University of Southern California (Viterbi) | 90 | 71 |  | 15 | Pennsylvania State University—University Park | 54 | 39 |
|  | 16 | Carnegie Mellon University | 22 | 16 |  | 14 | Texas A&M University--College Station | 57 | 32 |  | 16 | University of California--San Diego (Jacobs) | 60 | 51 |  | 15 | University of California—Los Angeles (Samueli) | 42 | 33 |
|  | 16 | Purdue University--West Lafayette | 33 | 21 |  | 17 | University of California--Los Angeles (Samueli) | 25 | 16 |  | 16 | University of Maryland--College Park (Clark) | 57 | 41 |  | 17 | University of California—San Diego (Jacobs) | 53 | 29 |
|  | 18 | Cornell University | 21 | 16 |  | 17 | University of Minnesota--Twin Cities | 28 | 17 |  | 16 | University of Wisconsin--Madison | 43 | 33 |  | 17 | Texas A&M University—College Station | 66 | 49 |
|  | 18 | University of Pennsylvania | 25 | 14 |  | 17 | University of Wisconsin--Madison | 33 | 25 |  | 16 | Virginia Tech | 86 | 52 |  | 17 | Columbia University (Fu Foundation) | 17 | 13 |
|  | 20 | Johns Hopkins University (Whiting) | 21 | 15 |  | 20 | Columbia University (Fu Foundation) | 17 | 11 |  | 20 | Texas A&M University--College Station | 71 | 42 |  | 17 | University of Pennsylvania | 30 | 20 |
| **Bottom 20** | 73 | University of Tennessee--Knoxville (Tickle) | 19 | 9 |  | 84 | Oklahoma State University | 17 | 6 |  | 85 | Stevens Institute of Technology (Schaefer) | 21 | 7 |  | 77 | University of California--Riverside (Bourns) | 23 | 15 |
|  | 73 | Washington State University | 22 | 8 |  | 84 | Stevens Institute of Technology (Schaefer) | 18 | 6 |  | 85 | Texas Tech University (Whitacre) | 25 | 5 |  | 83 | University of Cincinnati | 24 | 7 |
|  | 73 | Wayne State University | 16 | 8 |  | 84 | Stony Brook University--SUNY | 10 | 6 |  | 85 | University of Kansas | 32 | 8 |  | 83 | Clarkson University | 24 | 10 |
|  | 73 | Worcester Polytechnic Institute | 12 | 6 |  | 84 | University of Hawaii--Manoa | 18 | 12 |  | 85 | University of Texas--Arlington | 26 | 14 |  | 83 | Illinois Institute of Technology (Armour) | 27 | 17 |
|  | 88 | Clarkson University | 14 | 3 |  | 84 | University of Missouri | 19 | 9 |  | 85 | Worcester Polytechnic Institute | 19 | 5 |  | 83 | Stevens Institute of Technology (Schaefer) | 22 | 12 |
|  | 88 | Florida A&M University - Florida State University | 17 | 5 |  | 84 | University of New Mexico | 16 | 9 |  | 92 | Kansas State University | 16 | 4 |  | 83 | University of Houston (Cullen) | 27 | 17 |
|  | 88 | Kansas State University | 14 | 4 |  | 84 | University of South Carolina | 20 | 8 |  | 92 | Michigan Technological University | 19 | 4 |  | 83 | University of Kentucky | 36 | 14 |
|  | 88 | Missouri University of Science & Technology--Rolla | 12 | 2 |  | 84 | University of Wisconsin--Milwaukee | 11 | 5 |  | 92 | New Jersey Institute of Technology | 30 | 9 |  | 83 | University of Nebraska--Lincoln | 35 | 19 |
|  | 88 | New Jersey Institute of Technology | 12 | 3 |  | 95 | George Mason University (Volgenau) | 16 | 10 |  | 92 | Temple University | 12 | 5 |  | 83 | University of South Carolina | 36 | 12 |
|  | 88 | Oklahoma State University | 12 | 3 |  | 95 | Illinois Institute of Technology (Armour) | 13 | 8 |  | 92 | University of North Carolina--Charlotte (Lee) | 29 | 7 |  | 83 | University of Texas--Arlington | 39 | 21 |
|  | 88 | Syracuse University | 17 | 9 |  | 95 | Mississippi State University (Bagley) | 10 | 3 |  | 92 | University of Oklahoma | 27 | 10 |  | 91 | Louisiana State University--Baton Rouge | 22 | 9 |
|  | 95 | Rochester Institute of Technology (Gleason) | 7 | 5 |  | 95 | New Mexico State University | 16 | 6 |  | 92 | University of South Florida | 27 | 6 |  | 91 | New Jersey Institute of Technology | 28 | 13 |
|  | 95 | Stevens Institute of Technology (Schaefer) | 12 | 4 |  | 95 | San Diego State University | 16 | 10 |  | 99 | Air Force Institute of Technology 1 | 27 | 5 |  | 91 | Oklahoma State University | 30 | 8 |
|  | 95 | University of Akron | 15 | 2 |  | 95 | Southern Methodist University (Lyle) | 8 | 4 |  | 99 | Binghamton University--SUNY (Watson) | 17 | 4 |  | 91 | Texas Tech University (Whitacre) | 26 | 10 |
|  | 95 | University of Alabama | 23 | 5 |  | 95 | University of Alaska--Fairbanks | 9 | 2 |  | 99 | Illinois Institute of Technology (Armour) | 22 | 10 |  | 91 | University of Alabama | 30 | 9 |
|  | 95 | University of Arkansas--Fayetteville | 14 | 4 |  | 95 | University of Miami | 11 | 6 |  | 99 | Louisiana State University--Baton Rouge | 23 | 6 |  | 91 | University of Kansas | 19 | 8 |
|  | 95 | University of Cincinnati | 18 | 6 |  | 95 | University of Nevada--Las Vegas (Hughes) | 18 | 11 |  | 99 | University of Cincinnati | 43 | 10 |  | 91 | University of Massachusetts--Lowell (Francis) | 27 | 16 |
|  | 95 | University of Rochester (Hajim) | 13 | 5 |  | 95 | University of Texas--Arlington | 32 | 17 |  | 99 | University of Kentucky | 29 | 11 |  | 91 | University of New Mexico | 14 | 3 |
|  | 95 | Virginia Commonwealth University | 13 | 4 |  | 95 | University of Vermont | 11 | 6 |  | 99 | University of Missouri | 38 | 13 |  | 91 | University of Oklahoma | 26 | 9 |
|  | 95 | West Virginia University (Statler) | 17 | 7 |  | 95 | Worcester Polytechnic Institute | 12 | 4 |  | 99 | University of South Carolina | 17 | 2 |  | 91 | University of Rochester (Hajim) | 24 | 14 |
