# Supplementary material for: A systematic exclusion induced by institutional ranking in engineering faculty hiring: Introducing a cycle of winners and losers
Source: PLoS One. 2022 Dec 1;17(12):e0275861. doi: 10.1371/journal.pone.0275861 (PMC9714811; doi:10.1371/journal.pone.0275861)
Supplement: S2 Table — (DOCX) [file pone.0275861.s002.docx]

**S2 Table. All Engineering program rankings and financial profiles.**

| **Rank** | **NAME** | **Total 2019 R&D Expenditure (in thousands)** | **Endowment  (in thousands) 2019** | **Undergraduate Enrollment  Full Time (2019)** | **Endowment/ Student (in thousands) 2019** | **Tuition 2019** | **Retention Rate 2019** | **Student Loan Default rate (2017)** | **Graduation Rate 2018** | **Acceptance Rate 2019** |
| --- | --- | --- | --- | --- | --- | --- | --- | --- | --- | --- |
| Top 20 | Massachusetts Institute of Technology | 1,009 | 17,443,750 | 11,363 | 1,535 | 53,450 | 99% | 1.16% | 94.2% | 6.7% |
|  | Stanford University | 1,204 | 27,699,834 | 16,144 | 1,716 | 52,857 | 99% | 0.60% | 94.4% | 4.3% |
|  | University of California-Berkeley | 802 | 2,143,756 | 39,950 | 54 | 11,442 | 97% | 1.78% | 91.4% | 16.3% |
|  | California Institute of Technology | 399 | 2,987,001 | 2,237 | 1,335 | 52,506 | 98% | 1.06% | 92.0% | 6.4% |
|  | Carnegie Mellon University | 359 | 1,983,602 | 14,180 | 140 | 55,816 | 97% | 0.67% | 88.8% | 15.4% |
|  | Purdue University-Main Campus | 663 | 2,539,232 | 39,210 | 65 | 9,208 | 91% | 2.25% | 80.6% | 59.8% |
|  | University of Michigan-Ann Arbor | 1,675 | 12,273,834 | 45,510 | 270 | 16,212 | 97% | 1.27% | 91.9% | 22.9% |
|  | Georgia Institute of Technology-Main Campus | 960 | 2,169,005 | 20,593 | 105 | 10,258 | 97% | 1.31% | 87.3% | 20.6% |
|  | University of California-San Diego | 1,353 | 799,000 | 37,316 | 21 | 11,442 | 93% | 1.52% | 85.9% | 31.5% |
|  | University of Illinois at Urbana-Champaign | 677 | 1,772,258 | 44,022 | 40 | 14,188 | 93% | 2.45% | 84.3% | 59.0% |
|  | Texas A & M University | 952 | 12,632,093 | 59,447 | 212 | 7,940 | 93% | 2.98% | 81.9% | 57.8% |
|  | Cornell University | 1,144 | 6,974,637 | 23,921 | 292 | 56,550 | 97% | 1.13% | 94.5% | 10.9% |
|  | University of Southern California | 909 | 5,739,565 | 41,497 | 138 | 57,256 | 96% | 1.28% | 92.4% | 11.4% |
|  | The University of Texas at Austin | 696 | 4,288,184 | 47,910 | 90 | 10,824 | 96% | 2.74% | 82.8% | 31.8% |
|  | Columbia University in the City of New York | 1,003 | 10,950,738 | 26,487 | 413 | 57,763 | 99% | 2.10% | 95.8% | 5.5% |
|  | University of California-Los Angeles | 1,306 | 2,255,362 | 42,767 | 53 | 11,442 | 97% | 1.04% | 90.5% | 12.3% |
|  | Johns Hopkins University^a^ | 2,917 | 6,275,939 | 14,907 | 421 | 55,350 | 97% | 1.93% | 93.0% | 11.2% |
|  | University of Pennsylvania | 1,506 | 14,649,761 | 22,318 | 656 | 51,156 | 98% | 1.40% | 95.5% | 7.7% |
|  | Northwestern University | 857 | 8,244,818 | 18,194 | 453 | 56,232 | 98% | 0.88% | 94.6% | 9.1% |
|  | University of Maryland-College Park^b^ | 1,096 | 691,300 | 36,267 | 19 | 8,824 | 95% | 2.45% | 86.3% | 44.2% |
| Bottom 20 | University of Illinois at Chicago | 382 | 368,000 | 27,844 | 13 | 11,924 | 79% | 3.41% | 59.4% | 72.7% |
|  | Missouri University of Science and Technology | 41 | 201,200 | 6,612 | 30 | 9,120 | 82% | 3.19% | 62.7% | 79.2% |
|  | University of Iowa | 508 | 1,578,933 | 26,664 | 59 | 8,073 | 86% | 3.59% | 73.2% | 82.6% |
|  | University of New Mexico-Main Campus | 239 | 472,000 | 15,684 | 30 | 6,299 | 77% | 12.80% | 49.3% | 94.3% |
|  | The University of Texas at Arlington | 123 | 162,500 | 23,816 | 7 | 8,624 | 75% | 5.82% | 49.3% | 82.9% |
|  | Illinois Institute of Technology | 34 | 239,900 | 5,616 | 43 | 47,299 | 88% | 2.93% | 72.5% | 60.2% |
|  | Michigan Technological University | 80 | 117,624 | 6,264 | 19 | 16,834 | 84% | 2.34% | 68.4% | 74.3% |
|  | New Jersey Institute of Technology | 161 | 123,000 | 8,479 | 15 | 14,448 | 88% | 4.94% | 65.2% | 72.8% |
|  | University of California-Santa Cruz | 142 | 215,448 | 18,907 | 11 | 11,442 | 89% | 2.90% | 74.5% | 51.3% |
|  | University of Cincinnati-Main Campus | 529 | 624,728 | 29,466 | 21 | 9,476 | 88% | 7.20% | 67.0% | 76.7% |
|  | George Mason University | 186 | 113,000 | 26,081 | 4 | 9,060 | 86% | 2.64% | 70.0% | 86.7% |
|  | Mississippi State University | 264 | 529,000 | 18,951 | 28 | 8,800 | 82% | 8.36% | 58.3% | 53.9% |
|  | Naval Postgraduate School | 67 | **--** | **--** | **--** | **--** | **--** | **--** | -- | -- |
|  | University of Nebraska-Lincoln | 316 | 1,310,000 | 21,772 | 60 | 7,560 | 81% | 4.29% | 68.7% | 78.2% |
|  | Worcester Polytechnic Institute | 39 | 551,282 | 5,643 | 98 | 51,604 | 95% | 0.73% | 87.4% | 49.4% |
|  | Florida Agricultural and Mechanical University | 42 | 87,800 | 8,374 | 10 | 3,152 | 80% | 9.32% | 50.9% | 35.7% |
|  | Texas Tech University | 193 | 743,000 | 32,124 | 23 | 6,744 | 87% | 6.55% | 60.1% | 68.9% |
|  | University of South Florida | 391 | 480,000 | 31,973 | 15 | 4,559 | 91% | 4.57% | 73.4% | 47.6% |
|  | Brigham Young University | 39 | 1,880,000 | 30,119 | 62 | 5,790 | 90% | 1.73% | 86.4% | 67.5% |
|  | Embry Riddle Aeronautical University-Daytona Beach | 12 | 111,000 | 9,580 | 12 | 35,424 | 78% | 4.78% | 60.8% | 60.9% |
|  | ^a^ Johns Hopkins University includes the Applied Physics Laboratory, with $1,725,018 thousand in total R&D expenditures in FY 2019. | | | | | | | | | |
|  | ^b^ The University of Maryland includes expenditures from the University of Maryland, Baltimore and University of Maryland, College Park campuses. In FY 2019, the two campuses began reporting as one research unit to reflect their new strategic partnership. This relationship was codified through the University of Maryland Strategic Partnership Act passed by the Maryland General Assembly in 2016. Prior to FY 2019, both campuses reported to the survey as separate institutions. | | | | | | | | | |
|  | “--” Data not found | | | | | | | | | |
